# Increased dietary long-chain polyunsaturated fatty acids alter serum fatty acid concentrations and lower risk of urine stone formation in cats

**DOI:** 10.1371/journal.pone.0187133

**Published:** 2017-10-26

**Authors:** Jean A. Hall, Jeff A. Brockman, Stephen J. Davidson, Jen M. MacLeay, Dennis E. Jewell

**Affiliations:** 1 Department of Biomedical Sciences, College of Veterinary Medicine, Oregon State University, Corvallis, Oregon, United States of America; 2 Pet Nutrition Center, Hill's Pet Nutrition, Topeka, Kansas, United States of America; The University of Manchester, UNITED KINGDOM

## Abstract

The lifespan of cats with non-obstructive kidney stones is shortened compared with healthy cats indicating a need to reduce stone formation and minimize chronic kidney disease. The purpose of this study was to investigate the effects of increasing dietary polyunsaturated fatty acids (PUFA) on urine characteristics. Domestic-short-hair cats (n = 12; mean age 5.6 years) were randomized into two groups and fed one of two dry-cat foods in a cross-over study design. For one week before study initiation, all cats consumed control food that contained 0.07% arachidonic acid (AA), but no eicosapentaenoic acid (EPA) or docosahexaenoic acid (DHA). Group 1 continued eating control food for 56 days. Group 2 was fed test food for 56 days, which was control food plus fish oil and high-AA oil. Test food contained 0.17% AA, 0.09% EPA and 0.18% DHA. After 56 days, cats were fed the opposite food for another 56 days. At baseline and after each feeding period, serum was analyzed for fatty acid concentrations, and urine for specific gravity, calcium concentration, relative-super-saturation for struvite crystals, and a calcium-oxalate-titrimetric test was performed. After consuming test food, cats had increased (all *P*<0.001) serum concentrations of EPA (173%), DHA (61%), and AA (35%); decreased urine specific gravity (*P* = 0.02); decreased urine calcium concentration (*P* = 0.06); decreased relative-super-saturation for struvite crystals (*P* = 0.03); and increased resistance to oxalate crystal formation (*P* = 0.06) compared with cats consuming control food. Oxalate crystal formation was correlated with serum calcium concentration (*r* = 0.41; *P*<0.01). These data show benefits for reducing urine stone formation in cats by increasing dietary PUFA.

## Introduction

We recently reported that the lifespan of cats with kidney stones was on average 3 years shorter than that of cats without kidney stones. Thus, even non-obstructive kidney stones in cats affect the rate of chronic kidney disease (CKD) progression [1]. The majority of stones in cats are calcium oxalate or magnesium ammonium phosphate (struvite) uroliths. In 1985 there was a noticeable increase in the frequency of calcium oxalate uroliths in cats (55%), and a decrease in the frequency of struvite (33%) uroliths [2, 3]. The increase in frequency of calcium oxalate uroliths was thought to result from commercial food changes intended to decrease the recurrence of struvite uroliths and urethral plugs. This resulted in a reciprocal increase in the occurrence of calcium oxalate uroliths [4]. Whereas sterile struvite uroliths in cats can be readily dissolved in 1 to 4 weeks by feeding a food designed to promote formation of urine that is under-saturated with struvite [5, 6], it is generally accepted that medical dissolution is not possible for calcium oxalate uroliths [7]. Calcium oxalate stones are more likely to occur in older cats and in cats with CKD, although the relationship between the two is not fully understood. Calcium oxalate was the most common (88%) mineral composition noted in our previous study of cats with nephroliths [1].

Urine is a complex solution and is typically supersaturated with respect to stone forming substances [8]. In cats, calcium oxalate urolith formation occurs when urine is supersaturated with calcium and oxalate [9], although most healthy cats have urine that is supersaturated with calcium and oxalate. Achieving under-saturated urine with respect to calcium and oxalate is unlikely in cats, although food formulations can contribute to the formation of metastable urine, wherein crystals are less likely to form, and if they do form they are unlikely to grow. Various substances in the urine modify the process of stone formation. Inhibitors of calcium oxalate crystallization are molecules which interfere with crystal nucleation, aggregation, and growth. Inhibitors include citrate, magnesium, pyrophosphate, and organic molecules such as Tamm-Horsfall protein [8]. Substances that increase calcium oxalate crystallization are termed promoters, and include cellular debris, other types of crystals, urinary proteins, relatively low urine pH, calcium, sodium, oxalate, and urate [8]. Crystal formation is also enhanced by low urine volume or relatively slow flow of urine through the tubules. Ionic calcium and hypercalciuria can decrease inhibitory activity and promote crystallization. The role of cell injury may also be an important determinant in the promotion and progression of kidney stones by causing epithelial cell disruption and exposing nucleation sites within the kidney [8].

Although multiple inhibitors and promoters are involved in calcium oxalate crystallization, the effect of altering dietary composition as a means to prevent nephroliths is an area of active interest [10–12]. Arachidonic acid (AA) is an essential (n-6) fatty acid in cats and is the precursor for prostaglandin synthesis. Prostaglandins, in turn, are essential for maintaining vasodilation, and sodium and water balance in the kidney. Furthermore, sodium and calcium handling within the kidney are closely linked. Limited work suggests that dietary AA increases intestinal absorption of oxalate as well as increases clearance of oxalate in the kidneys [13]. Whereas the (n-6) fatty acids are involved in maintenance functions in the kidney, the (n-3) fatty acids (FA) may decrease calciuria and inhibit crystal formation, although their overall impact on urinary stone risk is uncertain [12]. The purpose of this study was to investigate the effects of added dietary polyunsaturated fatty acids (PUFA), including AA, eicosapentaenoic acid (EPA), and docosahexaenoic acid (DHA) on urine characteristics in healthy, adult cats.

## Materials and methods

### Animals and study design

All study protocols and this study were reviewed and approved by the Institutional Animal Care and Use Committee, Hill’s Pet Nutrition, Inc., Topeka, KS (Permit Number: CP607). Each cat had had a normal physical examination, complete blood count, serum chemistry profile, urinalysis, urine culture if indicated by the urinalysis results, blood taurine and total T4 concentrations measured within the 12 months prior to initiation of the study, or if not, blood and urine samples for these were collected during the screening phase and verified as normal. Inclusion criteria were healthy adult cats without evidence of concurrent disease. Cats also needed to be acclimated to single housing and the use of urine collection boxes with polypropylene beads. Cats with acute or chronic disease, abnormal physical examination, laboratory findings, urinalysis, or positive urine culture were excluded. No cats were removed from the study during the testing period.

Twelve healthy adult cats were fed one of two dry-cat foods for a four-month cross-over study. All cats were placed on a pre-trial food for 1 week, and then randomized into two groups for the study phase. During the study phase, cats consumed one food for 2 months and then were switched to the second food for the remainder of the study. All cats were domestic shorthair, and neutered. There were 2 males and 4 females in each group. At study initiation, cats in the control group were 6.2 years, 4 to 9 years (mean, range) with initial body weights of 4.29 ± 0.59 kg (mean ± SD). Cats in the treatment group were 5.0 years, 2 to 7 years (mean, range) with initial body weights of 4.93 ± 0.96 kg (mean ± SD). Complete blood count, serum chemistry profile, FA profile, urinalysis, urine protein to creatinine ratio, urine calcium oxalate titrimetric test (COT), struvite relative super saturation (RSS), and urine sediment analysis were assessed at initiation of the study and again at 1, 2, 3 and 4 months. Weekly body weights and water intakes, and daily food intakes were recorded.

All cats were individually housed and had access to natural light that varied with seasonal changes. All cats were provided with regular opportunities to exercise, with access to toys. All cats were owned by the commercial funders of this research or their affiliates, who gave permission for them to be included in this study.

Prior to this study, all cats had been fed many types of commercial and non-commercial foods of varying nutrient compositions, including dry and canned cat foods, in palatability studies. All foods met the requirements established by the Association of American Feed Control Officials for complete and balanced pet foods for adult cats.

### Foods

Food composition, expressed as percentage of food, as fed, is given in Table 1. Control food did not contain fish oil. Test food was control food plus added fish oil (0.7%) and added high-AA oil (0.2%) from algae (DSM, Heerlen, The Netherlands). The oils increased EPA, DHA, and AA FA as shown in Table 1. The concentrations of long-chain PUFA chosen for this study were based on previous studies [14, 15] whereby we showed that these concentrations would produce a significant increase in circulating fatty acids. Atwater energy was calculated using modified Atwater factors as previously described [16].

**Table 1:** Food composition.

Nutrient^a^	Control food^b^	Test food^c^
Moisture	5.15	5.11
Protein	35.78	34.16
Fat	15.81	16.70
Atwater Energy, kcal/kg	3,902	3,899
Ash	5.12	5.56
Crude Fiber	0.84	1.79
Calcium	0.80	0.84
Phosphorus	0.74	0.75
Sodium	0.34	0.36
Potassium	0.93	0.95
Magnesium	0.07	0.08
Lysine	1.45	1.45
Threonine	1.49	1.36
Methionine	0.95	0.95
Tryptophan	0.32	0.31
Lauric acid (12:0)	0.01	0.01
Myristic acid (14:0)	0.15	0.16
Palmitic acid (16:0)	3.28	3.38
Stearic acid (18:0)	1.42	1.45
LA (18:2; n-6)	2.75	2.72
αLA (18:3; n-3)	0.13	0.15
AA (20:4; n-6)	0.07	0.17
EPA (20:5; n-3)	<0.01	0.09
DPA (22:5; n-3)	<0.01	0.02
DHA (22:6; n-3)	<0.01	0.18
Σ SFA^d^	4.97	5.12
Σ MUFA^e^	6.15	6.27
Σ PUFA^f^	3.13	3.40
Σ (n-6) FA^g^	2.92	3.02
Σ (n-3) FA^h^	0.13	0.44
(n-6):(n-3) ratio	22.5	7.73

^a^ All analytical values are expressed as percentage of food, as fed, unless otherwise indicated.

^b^ Control food without added fish oil, Hill’s Pet Nutrition, Inc.

^c^ Test food was control food plus added fish oil (0.7%) and added high-AA oil (0.2%), Hill’s Pet Nutrition, Inc.

^d^ Sum of the saturated fatty acids: 8:0+10:0+11:0+12:0+14:0+15:0+16:0+17:0+18:0+20:0+22:0+24:0.

^e^ Sum of the monounsaturated fatty acids: 14:1+15:1+16:1+17:1+18:1+20:1+22:1+24:1.

^f^ Sum of the polyunsaturated fatty acids: 18:2(n-6)+18:3(n-6)+18:3(n-3)+18:4(n-3)+20:2(n-6)+20:3(n-6)+20:3(n-3)+20:4(n-6)+20:4(n-3)+20:5(n-3)+21:5(n-3)+22:2(n-6)+22:4(n-6)+22:5(n-6)+22:5(n-3)+22:6(n-3).

^g^ Sum of the (n-6) fatty acids.

^h^ Sum of the (n-3) fatty acids.

Fatty acid composition of the foods was determined by a commercial laboratory (Eurofins Scientific, Inc., Des Moines, IA) by gas chromatography of FA methyl esters. Fatty acid concentrations are expressed as g/100 g of FA as fed. The sum of dietary saturated fatty acids (SFA) was determined as follows: 8:0+10:0+11:0+12:0+14:0+15:0+16:0+17:0+18:0+20:0+22:0+24:0. The sum of dietary monounsaturated fatty acids (MUFA) was determined as follows: 14:1+15:1+16:1+17:1+18:1+20:1+22:1+24:1. The sum of dietary PUFA was determined as follows: 18:2(n-6)+18:3(n-6)+18:3(n-3)+18:4(n-3)+20:2(n-6)+20:3(n-6)+20:3(n-3)+20:4(n-6)+20:4(n-3)+20:5(n-3)+21:5(n-3)+22:2(n-6)+22:4(n-6)+22:5(n-6)+22:5(n-3)+22:6(n-3).

### Serum and urine analyses

Blood was collected from each cat (after withholding food for 17 h) at each time point (baseline and 1, 2, 3 and 4 months) to assess complete blood count and serum chemistries. Total T4 and blood taurine concentrations were assessed only at baseline to rule out underlying thyroid disease or deficiency. Similar to FA composition of foods, FA composition of serum samples was also determined by gas chromatography of FA methyl esters, with minor modifications [17] of the Folch et al. [18] method. Fatty acid concentrations in serum, determined using this methodology, were expressed as mg/dL.

Urine was collected at each assessment time over a 48-h period, into a bottle beneath the urine collection box containing polypropylene beads. Thymol was used to prevent microbial growth. Urine specimens were submitted for immediate analysis of urine pH, urine specific gravity (USG), routine dipstick analysis, semi-quantitative urine sediment analysis, and calculation of urine protein: creatinine (UPC) ratio. Urine specific gravity was determined using a refractometer. Urine creatinine concentration was used as an internal reference and measured with the same assay as serum creatinine. Urine calcium concentration was measured with the same assay as serum calcium. Urine protein concentrations were determined using urine supernatant (benzethonium chloride turbidometric method). The UPC ratio calculations were determined as previously reported [19] and expressed as mg/dL protein:mg/dL creatinine.

Concurrently, urine was analyzed for relative super saturation (RSS) for struvite crystals using the EQUIL 2 program [20–22]. In brief, this computer program calculates a urine supersaturation ratio (unitless) with respect to the common kidney stone components. The EQUIL 2 program provides an evaluation of the state of urinary saturation based on pH and total concentrations (M/L) of specific analytes. We measured sodium, potassium, calcium, magnesium, chloride, ammonium, citrate, phosphate, sulfate, and oxalate concentrations. The method uses thermodynamic stability constants to calculate free ion activities for urinary ions. These free ion activities are then used to calculate the supersaturation ratio of urine compared with what would form crystals in pure water.

A urine calcium oxalate titrimetric test (COT) was performed using a method adapted from Laube et al. [23–25]. In brief, the [Ca^+2^]/(added Ox^-2^) ratio is calculated (per liter), also termed the Bonn-Risk Index in humans. An increasing index value denotes samples at greater risk of calcium oxalate crystallization, whereas decreasing index values denotes those with less risk. The ratio represents the concentration of ionized calcium and the amount of oxalate that is added to initiate crystallization.

### Statistical analysis

The analyses for serum analytes and urinalysis parameters were performed using a repeated measures regression model (GLM in PROC MIXED) in Statistical Analysis Software version 9.4 (SAS Institute, Cary, NC) for effects of food, time, and food by time interaction. Animal was the experimental unit. Mean separation was performed using food formula and time on treatment as independent variables. All data are reported as least square means ± SEM. Significance was accepted as *P*<0.05, whereas *P*≤0.10 was considered a trend.

To investigate the independent variables affecting struvite RSS and oxalate crystal formation, we used a regression model (GLM in PROC MIXED) with animal as experimental unit. The interaction term between significant independent variables was used to calculate a linear function and the correlations between this term and RSS or oxalate crystal formation were then determined.

To investigate the relationship between resistance to oxalate crystal formation and serum calcium concentrations, Pearson correlation coefficients were measured using PROC GLM.

## Results

### The effect of dietary treatment on food and water intakes, body weights, and urine output

Water intakes of cats while consuming test food that contained increased concentrations of dietary PUFA were not consistently higher than those of cats consuming control food without extra PUFA. Adjusted water intakes were assessed, i.e., the individual intake minus the amount calculated for evaporation. After consuming test food, cats on average consumed 72.0 ± 5.8 g/day water, whereas cats consuming control food consumed 72.4 ± 5.8 g/day water. All cats were fed to maintain their body weights. Body weights did not change over the course of the study (data not shown). Urine output was 29.7 ± 5.0 mL/day for control cats and 30.3 ± 6.0 mL/day for cats consuming test food.

### The effect of dietary treatment on CBC, chemistry profiles, and FA profiles

No changes from physiologically normal concentrations were noted across time for CBC and chemistry profile data (data not shown). There were no treatment nor time effects on serum calcium concentrations (Table 2).

**Table 2:** Circulating concentrations of blood analytes and urinalysis parameters (mean ± SEM) of cats at baseline (initial) and after consuming control or test food for 28 and 56 days.

	Control food	Test food	Two-way ANOVA analysis		
			(*P* values)		
Number of animals, *n*	12	12	Food main effect	Time main effect	Food by time main effect
Circulating calcium and selected fatty acid concentrations:					
Calcium (mg/dL)					
Initial	9.9 ± 0.10	9.9 ± 0.10	0.80	0.80	0.05
28 days	10.1 ± 0.13	10.2 ± 0.13			
56 days	10.2 ± 0.13	10.1 ± 0.14			
Arachidonic acid (mg/dL)		
Initial	20.9 ± 1.7	20.9 ± 1.7	<0.001	0.01	0.84
28 days	19.1 ± 1.0^a^	26.5 ± 1.5^b^			
56 days	21.0 ± 1.3^a^	28.3 ± 1.5^b^			
EPA (mg/dL)					
Initial	1.24 ± 0.13	1.24 ± 0.13	<0.001	0.61	0.14
28 days	0.98 ± 0.10^a^	3.11 ± 0.22^b^			
56 days	0.85 ± 0.04^a^	3.39 ± 0.24^b^			
DHA (mg/dL)					
Initial	5.73 ± 0.49	5.73 ± 0.49	<0.001	0.88	0.60
28 days	4.70 ± 0.44^a^	9.08 ± 0.58^b^			
56 days	4.46 ± 0.47^a^	9.23 ± 0.58^b^			
Σ SFA^e^					
Initial	72.8 ± 2.84	72.8 ± 2.84	0.51	0.003	0.88
28 days	73.1 ± 2.47	75.0 ± 3.01			
56 days	79.5 ± 3.25	80.5 ± 3.91			
Σ MUFA^f^					
Initial	24.1 ± 0.89	24.1 ± 0.89	<0.001	0.002	0.84
28 days	25.0 ± 0.96^a^	20.3 ± 0.44^b^			
56 days	27.6 ± 0.90^a^	23.3 ± 1.24^b^			
Σ PUFA^g^					
Initial	79.8 ± 3.10	79.8 ± 3.10	0.88	0.001	0.34
28 days	79.2 ± 2.36	81.5 ± 3.42			
56 days	88.2 ± 3.94	87.4 ± 4.08			
Σ (n-6) FA^h^					
Initial	69.9 ± 2.57	69.9 ± 2.57	0.002	<0.001	0.22
28 days	70.7 ± 2.16^a^	66.8 ± 2.78^b^			
56 days	79.9 ± 3.57^a^	72.1 ± 3.46^b^			
Σ (n-3) FA^i^					
Initial	9.94 ± 0.71	9.94 ± 0.71	<0.001	0.71	0.45
28 days	8.48 ± 0.47^a^	14.7 ± 0.78^b^			
56 days	8.32 ± 0.60^a^	15.3 ± 0.84^b^			
Ratio of the Σ (n-6) to Σ (n-3) FA					
Initial	7.17 ± 0.26	7.17 ± 0.26	<0.001	0.05	0.11
28 days	8.62 ± 0.49^a^	4.59 ± 0.16^b^			
56 days	9.96 ± 0.69^a^	4.77 ± 0.84^b^			
Urinalysis parameters:					
pH					
Initial	5.97 ± 0.08	5.97 ± 0.08	0.26	0.36	0.84
28 days	6.47 ± 0.002	6.37 ± 0.002			
56 days	6.34 ± 0.002	6.19 ± 0.002			
Specific gravity					
Initial	1.050 ± 0.002	1.050 ± 0.002	0.02	0.26	0.41
28 days	1.057 ± 0.002^a^	1.052 ± 0.002^b^			
56 days	1.056 ± 0.002^a^	1.054 ± 0.002^b^			
Calcium (mg/dL)					
Initial	5.61 ± 0.80	5.61 ± 0.80	0.06	0.03	0.81
28 days	5.45 ± 0.71^c^	5.03 ± 0.50^d^			
56 days	4.96 ± 0.66^c^	4.42 ± 0.55^d^			
Fractional excretion of calcium (%)					
Initial	0.245 ± 0.037	0.245 ± 0.037	0.48	0.10	0.52
28 days	0.208 ± 0.031	0.205 ± 0.028			
56 days	0.193 ± 0.027	0.173 ± 0.017			
Struvite relative super saturation (unitless)					
Initial	3.5 ± 1.4	3.5 ± 1.4	0.03	0.18	0.24
28 days	12.6 ± 4.3^a^	5.2 ± 1.1^b^			
56 days	7.1 ± 1.4^a^	4.8 ± 1.1^b^			
Calcium oxalate titration test (1/L)					
Initial	65.5 ± 18.8	65.5 ± 18.8	0.06	0.53	0.94
28 days	56.7 ± 14.2^c^	42.8 ± 9.0^d^			
56 days	52.4 ± 12.2^c^	37.4 ± 8.5^d^			

^a,b^ Means with different superscripts within a row are different at *P*≤0.05.

^c,d^ Means with different superscripts within a row are different at *P*≤0.1.

^e^ Sum of the saturated fatty acids: 8:0+10:0+11:0+12:0+14:0+15:0+16:0+17:0+18:0+20:0+22:0+24:0.

^f^ Sum of the monounsaturated fatty acids: 14:1+15:1+16:1+17:1+18:1+20:1+22:1+24:1.

^g^ Sum of the polyunsaturated fatty acids: 18:2(n-6)+18:3(n-6)+18:3(n-3)+18:4(n-3)+20:2(n-6)+20:3(n-6)+20:3(n-3)+20:4(n-6)+20:4(n-3)+20:5(n-3)+21:5(n-3)+22:2(n-6)+22:4(n-6)+22:5(n-6)+22:5(n-3)+22:6(n-3).

^h^ Sum of the (n-6) fatty acids.

^i^ Sum of the (n-3) fatty acids.

After consuming test food, cats had increased (all *P*<0.001) serum concentrations of EPA (173%), DHA (61%), and AA (35%) compared with cats consuming control food (Table 2). Total SFA and total PUFA concentrations increased in cats consuming both control food and test food across time, whereas total MUFA concentrations increased in cats consuming control food, but decreased in cats consuming test food across time. Cats consuming test food had increased total (n-3) FA concentrations and decreased total (n-6) FA concentration compared with cats consuming control food, although (n-6) FA concentrations did increase in cats fed test food across time. The (n-6) and (n-3) FA concentrations were reflected in the ratio of total (n-6) to total (n-3) FA being 4.8 to 1.0 in cats fed test food and 10.0 to 1.0 in cats fed control food.

### The effect of dietary treatment on urinalysis parameters

There was not a significant effect of food or time on urine pH (Table 2). Urine pH was numerically higher in cats fed both foods compared with baseline, although the increase was smaller in cats fed test food. The range of urine pH in cats fed test food for 56 days was 5.5 to 7.5, whereas the range in cats fed control food for 56 days was 5.7 to 8.0.

The USG was lower (*P* = 0.02) at 28 and 56 days in cats while consuming test food containing increased concentrations of dietary PUFA compared with that of cats consuming control food without extra PUFA (Table 2).

Urine calcium concentration tended to decrease (*P* = 0.06; –21%) at 28 and 56 days in cats while consuming test food containing increased concentrations of dietary PUFA compared with that of cats consuming control food without extra PUFA (Table 2). There was also a time effect (*P* = 0.03) in that urine calcium concentration decreased in all cats across time.

The UPC ratios (all means, 0.1; data not shown) were not different between cats while consuming test food containing increased concentrations of dietary PUFA compared with cats consuming control food.

**Fig 1:**
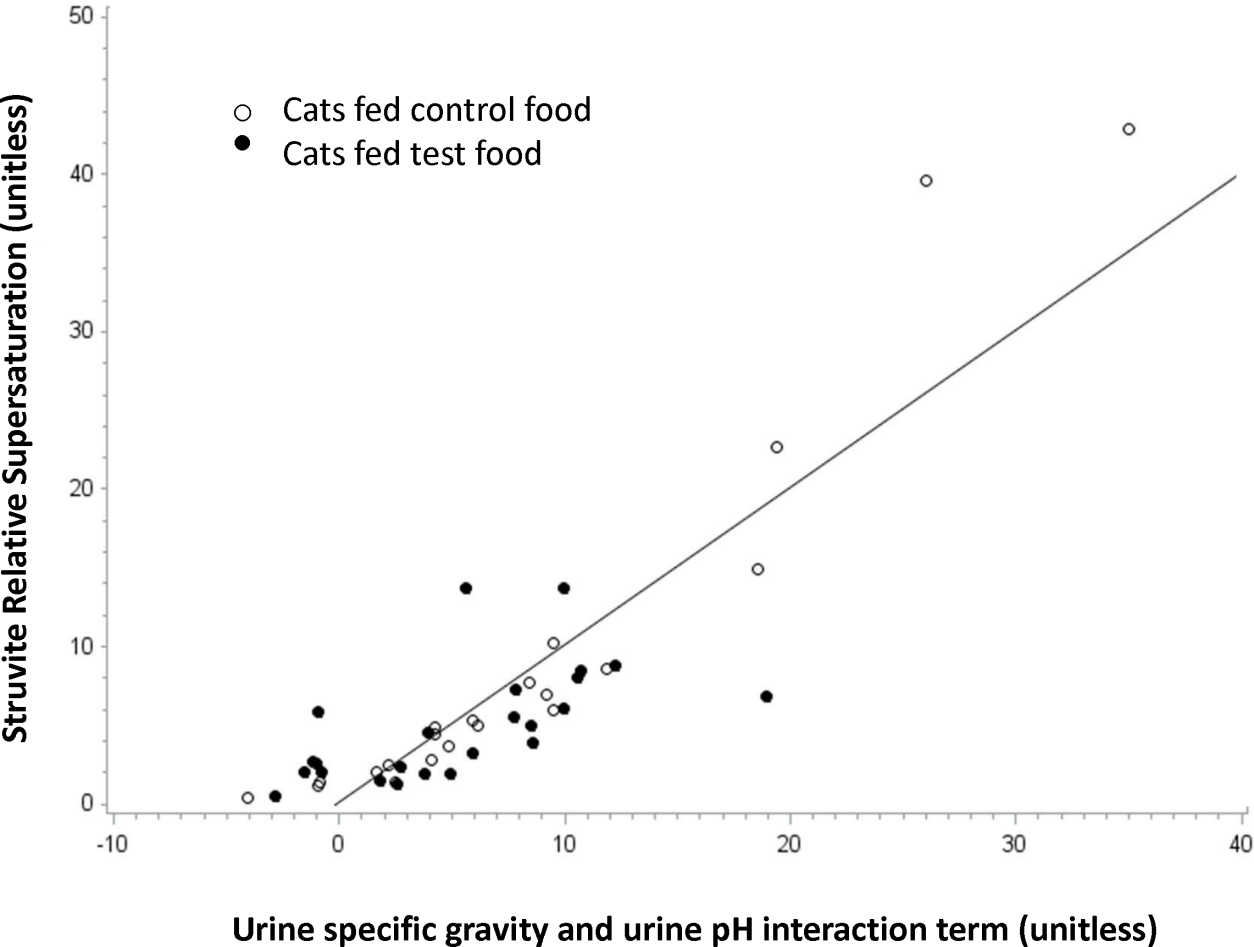
Struvite relative super saturation. The struvite relative super saturation (RSS; y-axis) is predicted using urine specific gravity (*P*<0.001) and urine pH (*P*<0.001) measurements (interaction *P*<0.001; x-axis). Each cat (*n* = 12) is represented twice by an open circle, for 28 and 56 days on control food, and twice by a filled circle, for 28 and 56 days on test food. Feeding test food decreased urine specific gravity and decreased urine pH, together both of which decreased struvite RSS (*r*2 = 0.69; *P*<0.001). Note that there are fewer cats fed test food in the upper right quadrant of the graph.

Struvite RSS values were lower (*P* = 0.03) in cats after consuming test food containing increased concentrations of dietary PUFA compared with those of cats consuming control food (Table 2), although all cats had higher scores across time than baseline values. At the end of the test food consumption period, struvite RSS values were 32% lower in cats after consuming test food compared with cats consuming control food. Using USG (*P*<0.001) and urine pH (*P*<0.001) measurements (interaction *P*<0.001), and animal as the experimental unit, we could predict struvite RSS. Feeding test food decreased USG and decreased urine pH, together both of which decreased struvite RSS (*r*2 = 0.69; *P*<0.001; Fig 1).

The urine calcium oxalate titrimetric test (COT) showed enhanced titratability to added oxalate before forming calcium oxalate crystals (*P* = 0.06; –43%) in cats while consuming test food containing increased concentrations of dietary PUFA compared with that of cats consuming control food (Table 2). Thus, feeding test food increased resistance to oxalate crystal formation. Also, oxalate crystal formation (COT) was positively correlated with serum calcium concentration (*r* = 0.41; *P*<0.01), and this relationship was unaffected by test food.

Urine sediment analysis revealed that the presence of crystals was higher for cats while consuming control food. Eleven of twelve cats experienced crystals while consuming control food. All cats had struvite (magnesium, ammonium, phosphate) crystals. One of the 11 cats had both calcium oxalate crystals and struvite crystals while consuming control food. Five cats experienced crystals while consuming test food (two cats in the first period and three cats in the second period). All five cats had struvite crystals.

**Fig 2:**
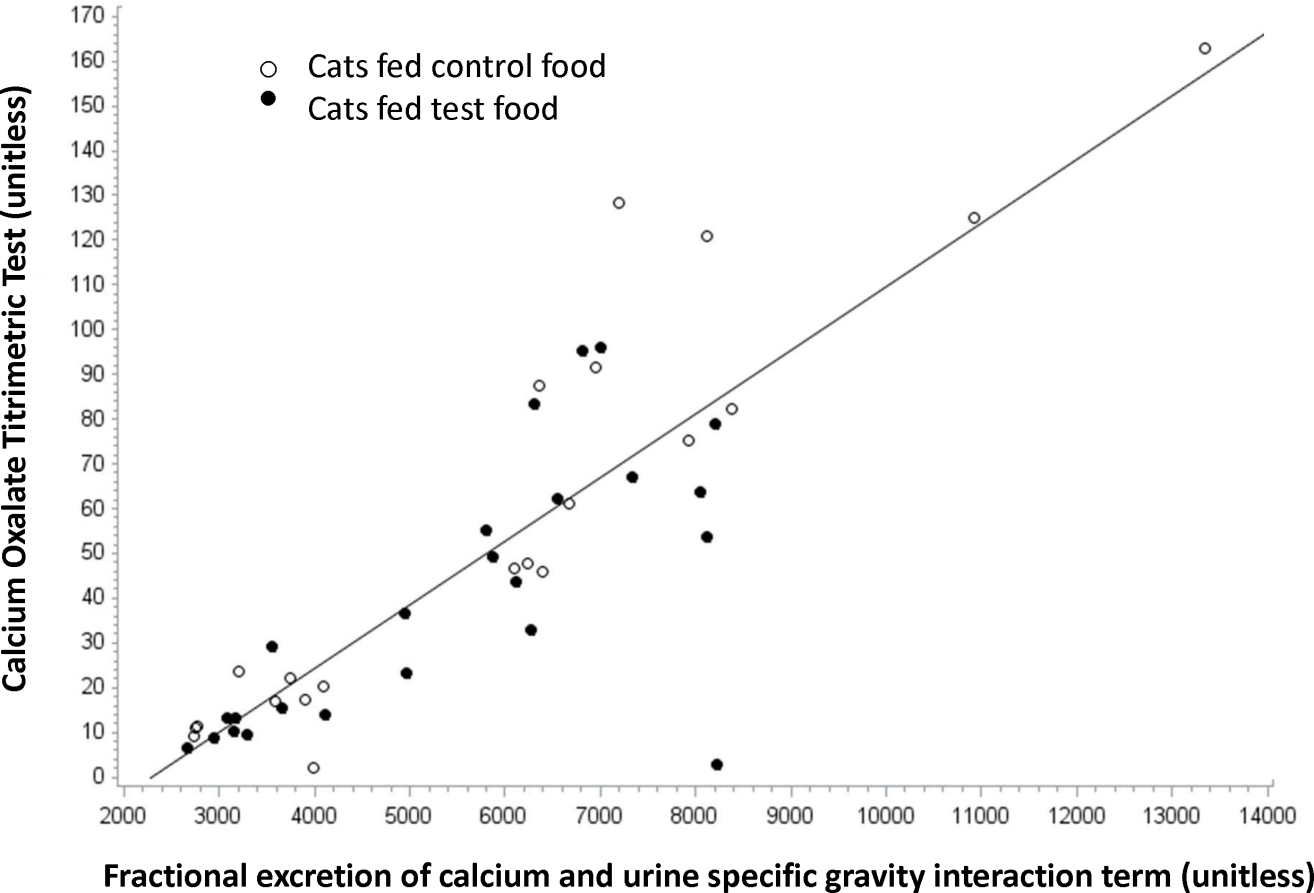
Calcium oxalate titrimetric test. The risk of oxalate crystal formation as determined by the calcium oxalate titrimetric test (COT; y-axis) is predicted using fractional excretion of calcium (*P* = 0.006) and urine specific gravity (*P* = 0.046) measurements (interaction *P* = 0.005; x-axis). Each cat (*n* = 12) is represented twice by an open circle, for 28 and 56 days on control food, and twice by a filled circle, for 28 and 56 days on test food. Feeding test food decreased fractional excretion of calcium and decreased urine specific gravity, together both of which increased resistance to oxalate crystal formation (*r*2 = 0.72; *P*<0.001). Note that there are fewer cats fed test food in the upper right quadrant of the graph.

Fractional excretion of calcium (%) was calculated as urine calcium concentration/serum calcium concentration divided by urine creatinine concentration/serum creatinine concentration × 100. There was not a significant effect of food or time on fractional excretion of calcium (Table 2). Fractional excretion of calcium was numerically decreased from baseline in cats fed both fed both foods, although the decrease was greater in cats fed test food. The range of fractional excretion values in cats fed test food for 56 days was 0.094 to 0.293%, whereas the range in cats fed control food for 56 days was 0.094 to 0.459%. Using fractional excretion of calcium (*P* = 0.006) and USG (*P* = 0.046) measurements (interaction *P* = 0.005), and animal as the experimental unit, we could predict risk of oxalate crystal formation (COT). Feeding test food decreased fractional excretion of calcium and decreased USG, together both of which increased resistance to oxalate crystal formation (*r*2 = 0.72; *P*<0.001; Fig 2).

## Discussion

Two nearly identical foods with regards to macronutrients, vitamins, and minerals were fed with the major difference being increased AA, EPA and DHA concentrations in the test food. Therefore, we could examine the impact of this combination of dietary PUFA on various urinary parameters. The hypothesis was that in the absence of major mineral differences between foods, the addition of these PUFA would provide evidence for a primary kidney health benefit. We found that dietary intervention with test food altered urine characteristic such that the RSS for struvite crystals was decreased (*P* = 0.03) and there tended to be increased resistance to oxalate crystal formation as determined by the calcium oxalate titrimetric test (COT; *P* = 0.06). These *in vitro* results were consistent with the corresponding *in vivo* urine sediment analyses, which revealed a greater presence of crystals in cats consuming control food compared with cats consuming test food. Furthermore, struvite RSS was predictable using USG and urine pH measurements (*P*<0.001; *r*2 = 0.69; Fig 1). Cats fed test food had decreased USG (*P* = 0.02) and decreased urine pH (numerically but not significantly) compared with cats fed control food, both of which decreased struvite RSS. The risk of oxalate crystal formation as determined by the COT assay was also predictable using fractional excretion of calcium and USG measurements (*P* = 0.005; *r*2 = 0.72; Fig 2). Cats fed test food had decreased fractional excretion of calcium (numerically but not significantly) and decreased USG (*P* = 0.02) compared with cats fed control food, both of which increased resistance to oxalate crystal formation. Previously, it was reported that cats fed food high in sodium chloride had increased fractional excretion of calcium and decreased urine specific gravity [26].

Dietary modification with increased PUFA resulted in decreased risk for struvite and calcium oxalate stone formation. Interestingly, the results of Lekcharoensuk et al. [4] suggest that it is unlikely that a nutrient change could result in lowering both struvite and calcium oxalate risk as the authors propose that risk for the two stone types is, broadly, a trade off; that is, when risk for one is high, the risk for the other is low. Never-the-less, in this study we saw a decrease in both RSS struvite and the COT test. We suggest that the overall impact of the combination of dietary PUFA on renal function results in relatively lower calcium excretion and relatively increased urinary water. Lower USG may have been the result of some combination of altered osmotic pressure in the collecting ducts, alterations in the renin/aldosterone system, and/or lower urine osmolality because of lower urine calcium. Arachidonic acid supplementation may have resulted in alterations in renal blood flow and/or Na-K-2Cl pump activity resulting in decreased urinary calcium; whereas increased (n-3) FA may have lowered urinary calcium excretion.

As expected, after consuming test food, cats had increased (all *P*<0.001) serum concentrations of EPA (173%), DHA (61%), and AA (35%). These FA results were anticipated based on previous findings in cats fed foods enriched with fish oil [14]. Cats make limited amounts of AA from linoleic acid presumably because they lack the Δ-6-desaturase enzyme [27], which makes AA an essential dietary FA in the cat. Therefore, additional AA (0.2%) was included in the test food. Added dietary AA in the test food did not affect absorption of or increases in serum EPA and DHA concentrations. We have also shown that the dose of (n-3) FA is more important than the dietary ratio of (n-6) to (n-3) FA in affecting the serum FA profile [28]. Consumption of test food resulted in a serum ratio of (n-6) to (n-3) FA of 4.8 to 1.0 versus 10.0 to 1.0 in cats fed control food.

It is well known that varying the FA composition of cell membranes with EPA and DHA from fish oil can affect many cellular functions, including membrane protein-mediated responses, lipid-mediator generation, cell signaling, and gene expression [29]. In general, the long-chain (n-3) PUFA are considered anti-inflammatory and immunomodulatory [30].

High urine calcium is known to promote calcium oxalate crystallization, and can arise from either increased calcium excretion or increased concentration of urine [8]. Control of hypercalciuria is the key to preventing biomineralization in the kidney, and is dependent upon physiological responses that load calcium in the blood as well as mechanisms within the kidney that result in calcium reabsorption or excretion in urine. In a review of the human literature, there is data to suggest that administration of (n-3) FA from fish oil alone, or combined with dietary counseling, decreases urinary calcium in hypercalcemic stone formers [12]. In one clinical study, Oriz-Alvarado et al. [31] studied the use of fish oil supplementation for dietary management of hypercalciuric stone formers. The use of (n-3) FA decreased urine calcium concentration in 52% of patients, urine oxalate excretion in 34% of patients, and calcium oxalate supersaturation in 38% of patients [31]. In another clinical study, patients with urinary stones were administered EPA daily for 18 months and urinary calcium was significantly reduced in the hypercalciuric group [32]. In rats, the impact of different dietary ratios of (n-6) and (n-3) FA showed that there was decreased urinary calcium excretion (42% after 6 weeks of supplementation) in the highest (n-3) supplementation group [33], demonstrating a negative relationship between EPA in the food and excretion of urinary calcium.

Certain populations have a low incidence of renal stones, e.g., Greenland Eskimos, which has been attributed to their food, which is rich in EPA [34]. This (n-3) FA is thought to have a protective role in preventing nephrolithiasis by decreasing urinary calcium and oxalate excretion through alteration of prostaglandin metabolism [34]. The role of AA in calciuria is unclear, and may be altered in individuals prone to hypercalciuria. Ionized calcium and calcium-containing salts are filtered at the glomerulus; blood flow and capillary pressure at the glomerulus are dependent upon prostaglandins. In turn, calcium reabsorption is highly regulated with approximately 98% of it being reabsorbed [35]. It has been suggested that increased phospholipid AA content may induce hypercalciuria by modulating calcium handling in intestinal, bone, and renal cells [36]. This is because enhanced PGE_2_ production, the main AA metabolite, can result in increased active vitamin D synthesis and modulate renal calcium reabsorption in the think ascending limb of Henle’s loop [36]. Dietary (n-3) PUFA have been shown to induce a significant decrease in PGE_2_ production in the kidney as well as its urinary excretion [37, 38]. Inhibition of PGE_2_ not only decreases urinary calcium excretion, but also leads to increased renal calcium reabsorption via its effect on nephron transporters [32]. In our study with healthy cats, we did not measure urinary prostaglandin excretion as an indicator of renal PG synthesis to know if PGE_2_ was decreased and correlated with hypocalciuria.

Siener et al. [39] evaluated the physiological effects of (n-3) FA supplementation with EPA and DHA on risk factors for calcium oxalate stone formation in healthy human subjects and found no effect on urinary calcium excretion. Irrespective, RSS with calcium oxalate was significantly decreased (23% after 30 days of supplementation) due to significantly decreased urinary oxalate excretion [39]. They hypothesized that an altered FA pattern of membrane phospholipids was associated with concomitant changes in oxalate transporter activity [39]. A specific modulatory effect of AA on erythrocyte oxalate transport has been demonstrated [36, 40]. Increased AA in membrane phospholipids may induce oxalate excretion into urine by activating anion carriers, e.g., renal transporters of oxalate [36].

In our study, predisposition to oxalate crystal formation determined by the COT assay was positively correlated with serum calcium concentration (*r* = 0.41; *P*<0.01). Serum calcium concentration was not affected by test food in these healthy cats. Thus, cats with higher serum calcium were at higher risk of oxalate crystal formation as determined by the COT assay. However, consuming test food lowered their predisposition to oxalate crystal formation by decreasing urinary calcium excretion.

The changes in USG in cats of this study fed test food compared with cats fed control food were minimal, yet significant. Both foods were dry cat foods. Water intakes of cats while consuming test food were similar to those of cats while consuming control food. Cats had increased USG compared with baseline values in both treatment and control groups, yet the increase was greater in cats fed control food (1.050 to 1.056 or 1.057) versus cats fed test food (1.050 to 1.052 or 1.054). Low urine volume is known to promote stone formation [8], so the smaller increase in USG in cats fed test food was important in decreasing risk for stone formation.

In human nephrolithiasis patients, regardless of the underlying cause of stone disease, a major goal of medical management is to increase daily fluid intake [12]. However, voluntary fluid intake is relatively easy to achieve in humans compared with cats. Ideally, increased fluid intake can benefit the patient through increased urine volume to prevent urine stagnation, formation of crystals, and growth of stones, but more importantly, dilute urine alters the supersaturation of stone components [12]. In a review of the human literature, there was no evidence showing actual benefit of increased water intake for first-time stone formers, but there was randomized controlled trial evidence of benefit for increasing water intake to prevent stone recurrences [12]. In cats, the only proven method to increase fluid intake is to feed foods with higher moisture content (74 to 81%) and an association between wet food feeding and decreased risk of stone formation has been shown. Cats fed wet foods were less likely to develop calcium oxalate uroliths compared with cats fed foods low in moisture (7 to 8%) [4]. However, transitioning cats to a 100% wet food regimen can be a challenge.

Based on evaluations of foods fed to cats with struvite (magnesium ammonium phosphate) uroliths, Lekcharoensuk et al. [4] suggested that foods designed to increase urine acidifying potential may decrease formation of struvite uroliths [4]. In support, magnesium ammonium phosphate is more soluble at low pH. Thus, it was not surprising in our study that cats fed test food, which resulted in decreased USG and numerically decreased urine pH relative to cats fed control food, had decreased struvite RSS. The urine pH of cats consuming test food was not significantly different compared with cats consuming control food. However, this decrease was enough to allow urine pH to play a significant predictive role in the struvite RSS assay. It is known that medical dissolution of sterile struvite uroliths in cats involves increasing the solubility of crystalloids in urine by changing urine pH to a less favorable environment for crystallization (≤ 6.3 pH) [41]. Under-saturation of urine with calculogenic crystalloids is achieved by lowering urine osmolality, by increasing the volume of urine in which crystalloids are dissolved or suspended, and by reducing the quantity of calculogenic crystalloids in urine by changing the food [41].

Limitations of this research are the relatively small group of healthy cats used in this study. Studies in cats with nephroliths are needed to determine COT values and whether those measurements can be decreased by increasing dietary PUFA. Further clinical studies are planned to compare addition of (n-6) or (n-3) fatty acids individually, to differentiate the effects produced by each on RSS and COT values. Finally, prospective clinical studies are needed to determine whether PUFA supplementation is effective for preventing calcium oxalate stone formation in cats.

## Conclusions

These data show benefits for reducing urine stone formation in cats by increasing dietary PUFA. There was both a decrease in RSS for struvite crystals and an increase in resistance to oxalate crystal formation in cats consuming test food compared with cats consuming control food. These *in vitro* test results were consistent with the *in vivo* urine sediment analysis which revealed that the presence of urine crystals was higher for cats while consuming control food compared with cats consuming test food.

## Abbreviations

AA: arachidonic acid
COT: calcium oxalate titrimetric test
CKD: chronic kidney disease
DHA: docosahexaenoic acid
EPA: eicosapentaenoic acid
FA: fatty acids
MUFA: monounsaturated fatty acids
PUFA: polyunsaturated fatty acids
RSS: relative super saturation
SFA: saturated fatty acids
UPC: urine protein: creatinine
USG: urine specific gravity
